# Chikungunya Fever in Traveler from Angola to Japan, 2016

**DOI:** 10.3201/eid2301.161395

**Published:** 2017-01

**Authors:** Saho Takaya, Satoshi Kutsuna, Eri Nakayama, Satoshi Taniguchi, Shigeru Tajima, Yuichi Katanami, Kei Yamamoto, Nozomi Takeshita, Kayoko Hayakawa, Yasuyuki Kato, Shuzo Kanagawa, Norio Ohmagari

**Affiliations:** National Center for Global Health and Medicine, Tokyo, Japan (S. Takaya, S. Kutsuna, Y. Katanami, K. Yamamoto, N. Takeshita, K. Hayakawa, Y. Kato, S. Kanagawa, N. Ohmagari);; National Institute of Infectious Diseases, Tokyo (E. Nakayama, S. Taniguchi, S. Tajima)

**Keywords:** chikungunya fever, yellow fever, arboviruses, Angola, Africa, viruses

## Abstract

Simultaneous circulation of multiple arboviruses presents diagnostic challenges. In May 2016, chikungunya fever was diagnosed in a traveler from Angola to Japan. Travel history, incubation period, and phylogenetic analysis indicated probable infection acquisition in Angola, where a yellow fever outbreak is ongoing. Thus, local transmission of chikungunya virus probably also occurs in Angola.

Simultaneous circulation of multiple arboviruses has been observed several times in many parts of the world. In 1970, Angola reported an outbreak of a dengue-like syndrome, which turned out to be a concurrent outbreak of yellow fever and chikungunya fever (*1*). On April 13, 2016, the World Health Organization declared a yellow fever outbreak in Angola. In response to the outbreak, a nationwide yellow fever vaccination campaign was initiated. As of July 29, 2016, a total of 3,818 confirmed and suspected cases were reported (*2*). In addition, on July 23, 2016, the World Health Organization was notified of a Rift Valley fever case in a man from China working in Luanda, the capital city of Angola, and started an investigation in Angola (*3*). We describe a case of chikungunya fever in a traveler from Angola to Japan.

In May 2016, a 21-year-old woman traveled to Tokyo, Japan, from her home in Luanda. She began to exhibit a high fever on the first day of her visit. On the second day, she sought care at the National Center for Global Health and Medicine (Tokyo). She had been previously healthy and had not traveled out of Luanda in the past 6 months. She claimed to have been vaccinated according to the national immunization plan, which included vaccination against yellow fever. At the first visit, she had high-grade fever (40.7°C) without other signs. Her vital signs were otherwise stable, and physical examination revealed no abnormality. Complete blood count and biochemistry tests revealed only a slightly elevated C-reactive protein level (1.55 mg/dL). Results of rapid diagnostic testing for malaria and dengue, 3 consecutive thin blood smears, HIV screening, and blood culture for bacteria were all negative.

After hospitalization, her fever gradually subsided but remained above 38°C. On the fifth day, bilateral axillary lymphadenopathy appeared. The lymph nodes were ≈2 cm, painful, and nonfluctuant. Despite the high-grade fever and lymphadenopathy, her general condition improved, and she was discharged on the fifth day. Thereafter, she recovered quickly and returned safely to Luanda. 

Although the patient was supposedly vaccinated against yellow fever virus, we performed real-time reverse transcription PCR for yellow fever virus, and the result was confirmed to be negative. Testing for other arboviruses was performed, and real-time reverse transcription PCR for chikungunya virus (CHIKV) showed a positive result. Therefore, the final diagnosis was chikungunya fever. We used phylogenetic analysis based on the nucleotide sequence of the E1 gene from the serum sample, the maximum-likelihood method with 1,000 bootstrap replicates, and MEGA 6.0 software (http://www.megasoftware.net). The sequence was 98% identical to that of a CHIKV strain isolated in the Central African Republic in 1987 (Figure). Considering travel history, incubation period, and phylogenetic analysis, the patient was probably infected with CHIKV while in Luanda.

**Figure:**
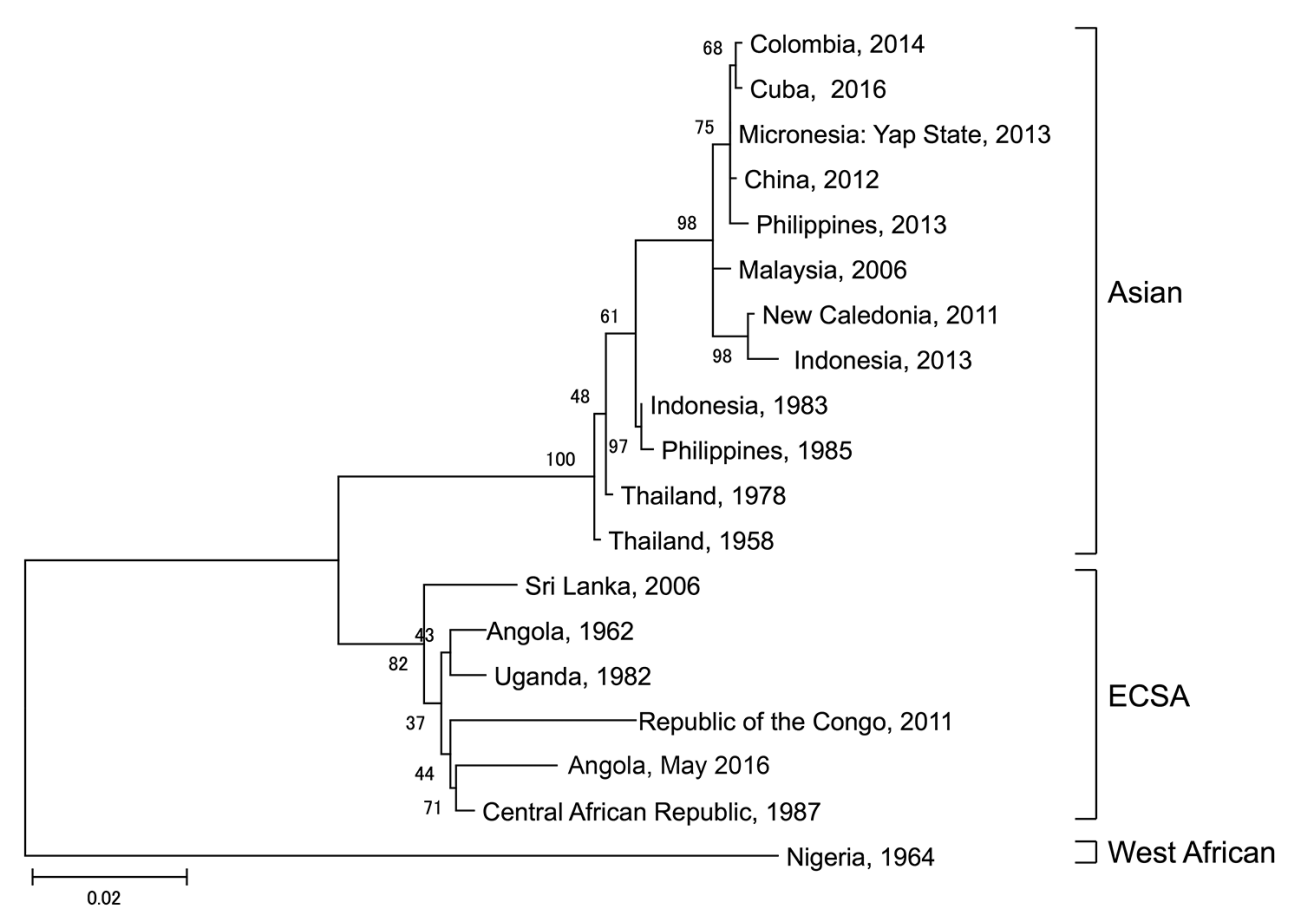
Phylogenetic comparison of the chikungunya virus sequence obtained from a patient traveling from Angola to Japan in May 2016 and reference sequences. Virus lineages are shown on the right. Scale bar represents substitutions per nucleotide position. ECSA, East/Central/South African lineage.

CHIKV was first isolated in Tanzania in 1953 (*4*). After a few decades of absence in Africa, the virus caused a large outbreak in the Democratic Republic of the Congo in 2000 (*5*) and has subsequently been causing infection across the continent. Although the epidemiology of chikungunya fever is scarcely understood in Africa, an effort has been made to grasp the current burden of CHIKV in Africa. A study in Kenya found the rate of CHIKV IgG positivity among HIV-negative specimens to be 0.96% (*6*). A serologic study in southern Mozambique found that the rate of seroconversion or a >4-fold titer rise of CHIKV IgG among patients with acute febrile illness was 4.3% (*7*). These studies suggest that the incidence of CHIKV infection in Africa may be higher than previously assumed. This discrepancy may be explained by lack of awareness, diagnostic tools, and surveillance systems. As of April 22, 2016, Angola was not recognized as a country with local CHIKV transmission (*8*). However, considering that Angola harbors *Aedes aegypti* mosquitoes, which are efficient CHIKV vectors, and that neighboring countries have documented local transmission of the virus, it is reasonable to speculate that local transmission also occurs in Angola.

Co-infection and co-distribution of multiple arboviruses (including dengue viruses, CHIKV, and yellow fever virus) are widely reported (*1**,**9**,**10*). Although these viruses share a common vector, *Aedes* spp. mosquitoes, their interactions within mosquitoes and their effects on vector competence are unknown (*9*). Arboviruses cause similar clinical presentations, which makes diagnosis challenging without labor-intensive diagnostics, especially in outbreak settings. Because a yellow fever outbreak is ongoing in Angola, the diagnosis of other arboviral infections is needed for conducting appropriate clinical and public health interventions and precise surveillance.

This case highlights 2 issues: the unknown epidemiology of CHIKV in Africa and the difficulty of diagnosing one arboviral infection during an outbreak of another arboviral infection. Further research is necessary to elucidate the true extent of CHIKV in African countries and to understand the public health implications of co-infection and co-distribution of multiple arboviruses.
